# Supplementary material for: The interactome of intact mitochondria by cross-linking mass spectrometry provides evidence for coexisting respiratory supercomplexes
Source: Mol Cell Proteomics. 2017 Dec 8;17(2):216–32. doi: 10.1074/mcp.RA117.000470 (PMC5795388; doi:10.1074/mcp.RA117.000470)
Supplement: Supplemental Data [file supp_RA117.000470_133922_0_supp_23978_fzffwf.zip › spectra_annotation/mito_spectra_annotation/123-1-2-1-8-1.pdf]

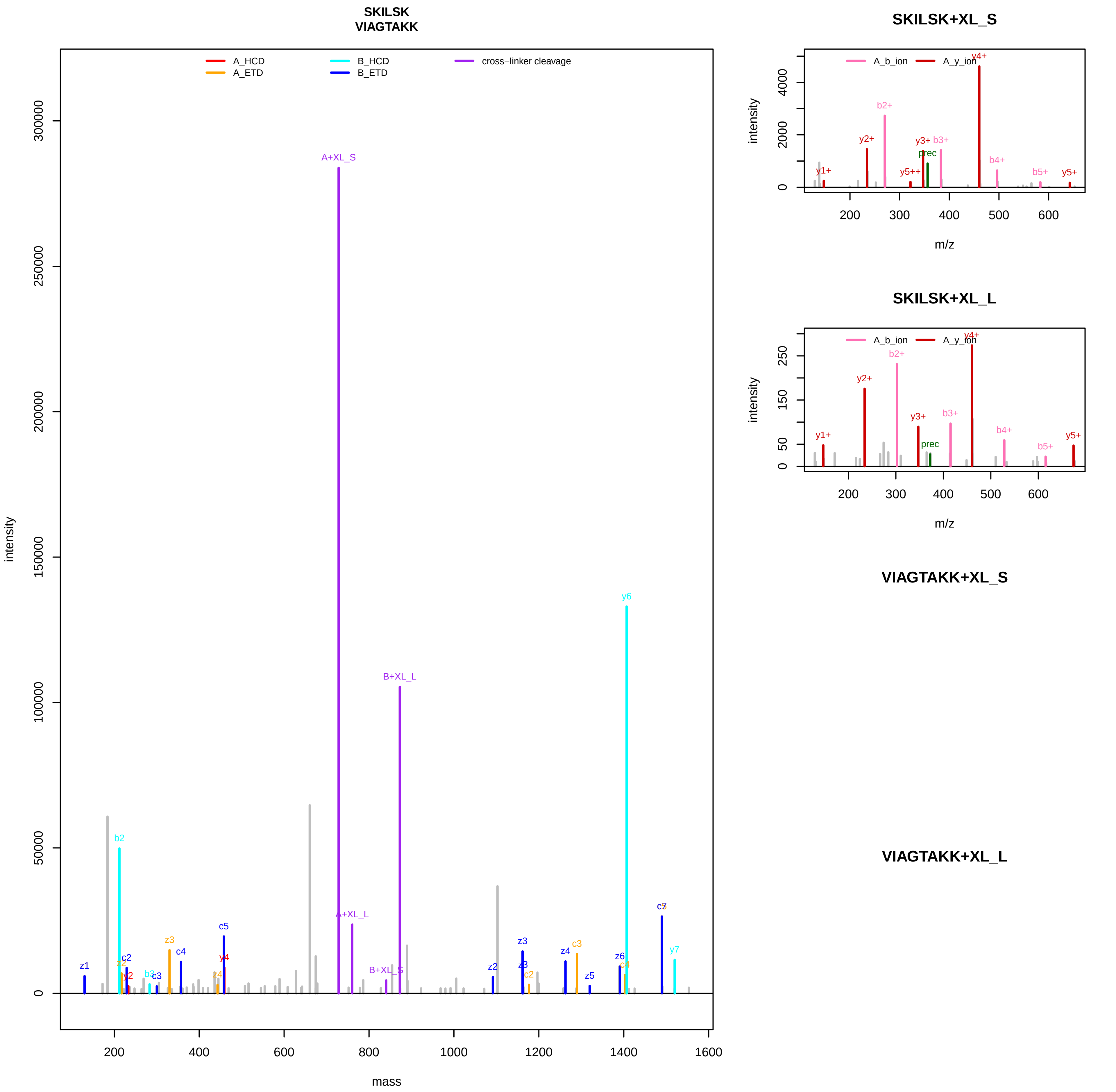
SKILSK
VIAGTAKK
A_HCD
A_ETD
B_HCD
B_ETD
cross−linker cleavage
A+XL_S
B+XL_L
A+XL_L
B+XL_S
y6
y7
b2
b3
c2
c3
c4
c5
c7
z1
z2
z3
z4
z5
z6
y2
y4
intensity
mass
SKILSK+XL_S
SKILSK+XL_L
A_b_ion
A_y_ion
y1+
y2+
y3+
y4+
y5+
y5++
b2+
b3+
b4+
b5+
prec
m/z
VIAGTAKK+XL_S
VIAGTAKK+XL_L
